# Implementation of Highly Stable Memristive Characteristics in an Organic–Inorganic Hybrid Resistive Switching Layer of Chitosan-Titanium Oxide with Microwave-Assisted Oxidation

**DOI:** 10.3390/molecules28135174

**Published:** 2023-07-03

**Authors:** Dong-Hee Lee, Hamin Park, Won-Ju Cho

**Affiliations:** 1 Department of Electronic Materials Engineering, Kwangwoon University, Gwangun-ro 20, Nowon-gu, Seoul 01897, Republic of Korea; zpsxlzje@naver.com; 2 Department of Electronic Engineering, Kwangwoon University, Gwangun-ro 20, Nowon-gu, Seoul 01897, Republic of Korea; parkhamin@kw.ac.kr

**Keywords:** microwave, organic–inorganic hybrid memristor, biocompatible, chitosan, analog switching, synaptic weight change, neuromorphic computing system

## Abstract

This study proposes a high-performance organic–inorganic hybrid memristor for the development of neuromorphic devices in the memristor-based artificial synapse. The memristor consists of a solid polymer electrolyte (SPE) chitosan layer and a titanium oxide (TiO_x_) layer grown with a low-thermal-budget, microwave-assisted oxidation. The fabricated Ti/SPE–chitosan/TiO_x_/Pt-structured memristor exhibited steady bipolar resistive switching (BRS) characteristics and demonstrated excellent endurance in 100-cycle repetition tests. Compared to SPE–chitosan memristors without a TiO_x_ layer, the proposed organic–inorganic hybrid memristor demonstrated a higher dynamic range and a higher response to pre-synaptic stimuli such as short-term plasticity via paired-pulse facilitation. The effect of adding the TiO_x_ layer on the BRS properties was examined, and the results showed that the TiO_x_ layer improved the chemical and electrical superiority of the proposed memristor synaptic device. The proposed SPE–chitosan organic–inorganic hybrid memristor also exhibited a stable spike-timing-dependent plasticity, which closely mimics long-term plasticity. The potentiation and depression behaviors that modulate synaptic weights operated stably via repeated spike cycle tests. Therefore, the proposed SPE–chitosan organic–inorganic hybrid memristor is a promising candidate for the development of neuromorphic devices in memristor-based artificial synapses owing to its excellent stability, high dynamic range, and superior response to pre-synaptic stimuli.

## 1. Introduction

Current state-of-the-art computing systems face enormous challenges when dealing with large amounts of unstructured data and real-time decision-making processes because of the von Neumann bottleneck limitations [1,2], which have necessitated the development of new intelligent computing platforms that can overcome these limitations. One such platform is the two-terminal metal-insulator-metal (MIM) structure memristor, which offers several advantages such as simplicity, nonvolatile memory, and computations using consecutive analog resistive switching (RS) in the insulator layer [3,4,5]. Several studies have investigated the development of computing systems capable of performing complex computations based on analog RS. The synaptic plasticity RS layer of memristors has been extensively studied using various materials, including inorganic, organic, and hybrid nanocomposites [6,7]. Numerous studies have reported on RS behaviors of bio-inspired organic materials, including chitosan, cellulose, albumen, and gelatin [8,9,10]. For compatibility with advanced biocompatible electronic devices, such as wearable devices that require high flexibility, stretchability, and transparency, potential materials must be low-temperature, processable, natural, organic materials based on a solution state, which offer diverse engineering platforms and are viable alternatives to inorganic-based solutions owing to their biodegradability, non-toxicity, biocompatibility, and bio-absorbability [6,11]. Among the various bio-inspired natural organic materials, chitosan electrolytes have several advantages suitable for solid polymer electrolyte (SPE)-based memristor devices. Firstly, chitin, the main ingredient of chitosan, is the second most abundant polysaccharide after cellulose. Secondly, chitosan’s amine and hydroxyl groups are particularly reactive with metal ions. Thirdly, although it naturally is an insulator, the ion conductivity can be modulated by adding an acidic solution. Fourthly, chitosan powder can be easily dissolved in a diluted acetic acid solution. Therefore, a thin film made from chitosan, which has low-cost solution processability, exhibits remarkable flexibility and transparency [12,13,14]. However, the low endurance and unstable retention of bio-organic-based memristors must be resolved. Therefore, RS layers using hybrid nanocomposites have been actively investigated in recent years [15,16]. The first report of chemical reactions via microwave irradiation (MWI) dates back to 1986 [17]. MWI induces friction and rotation of polar molecules, enabling direct and uniform internal heating, which has been considered an eco-friendly and high-efficiency heating method. MWI is effective in the manufacturing of various materials and in organic synthesis [18,19]. Furthermore, MWI provides more benefits than traditional heat treatment methods as it can selectively heat samples with a high heat transfer efficiency, a short processing time, low energy consumption, and cost-effectiveness [20,21,22].

In this study, we employed a low-thermal-budget microwave (MW)-assisted oxidation method to fabricate a titanium oxide (TiO_x_) layer, which has high carrier mobility and inherent chemical stability [23]. Inspired by the unique characteristics of chitosan and TiO_x_, we fabricated solid polymer electrolyte (SPE)-chitosan memristors with TiO_x_ and evaluated their endurance in RS behavior and resistance distribution, as well as their artificial synaptic behaviors. To verify the efficiency of MW-assisted oxidation, we prepared an RS layer without TiO_x_ for comparison. We evaluated the bipolar resistive switching (BRS) operation and memristive switching properties of the proposed devices. Additionally, we analyzed the short- and long-term plasticity for crucial artificial synaptic behaviors such as paired-pulse facilitation (PPF), spike-timing-dependent plasticity (STDP), and potentiation/depression. The results demonstrated the feasibility of using TiO_x_-based SPE–chitosan memristors as efficient and reliable synaptic devices for electronic synaptic systems.

## 2. Materials and Methods

### 2.1. Materials

The materials used to fabricate two-terminal memristors in this study included p-type (100) Si wafers with a resistivity range of 1–10 Ω·cm purchased from LG SILTRON Inc. (Gumi, Republic of Korea), Pt pellets (purity, >99.95%) and Ti pellets (purity, >99.99%) purchased from TIFINE Co. (Seoul, Republic of Korea), and chitosan powder (deacetylation degree, >75%) and acetic acid solution (purity > 99%) purchased from Sigma-Aldrich (Seoul, Republic of Korea).

### 2.2. Preparation of the Chitosan Solution

For the preparation of a biocompatible chitosan solution, a mixture of chitosan powder derived from shrimp shells and acetic acid solution was used. Specifically, 2 wt% chitosan powder was added to a 2 wt% acetic acid solution, which was then diluted with 10 mL of deionized water. The mixture was stirred constantly at 800 rpm for 6 h at 50 °C using a magnetic stirrer until the powder entirely dissolved. To remove various impurities, the solution was filtered using a polytetrafluoroethylene syringe filter with a pore size of 5 μm (Whatman International Ltd., Maidstone, UK).

### 2.3. Fabrication of the SPE–Chitosan Memristor with TiO_x_ through MW-Assisted Oxidation

**Figure 1:**
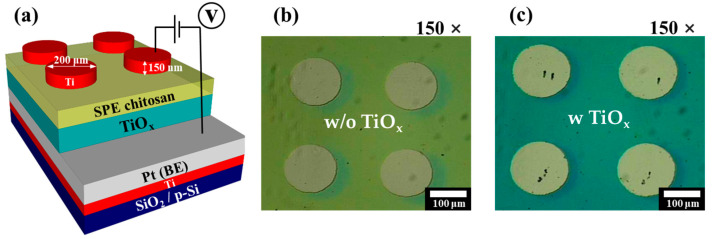
(**a**) Schematic diagram of a SPE–chitosan memristor with an embedded TiO_x_ layer developed with MW-assisted oxidation. Optical microscopy images of SPE–chitosan memristor without TiO_x_ (**b**) and with TiO_x_ (**c**) at a magnification of 150×.

We fabricated highly stable SPE–chitosan memristors with an embedded TiO_x_ layer through MW-assisted oxidation. The process started with cleaning the p-type Si wafer ((110) planes silicon wafers) with a 300 nm-thick thermally grown oxide using a standard Radio Corporation of America (New York, NY, USA) cleaning method. To form the bottom electrode (BE) of the memristor with a MIM structure, a 10 nm-thick Ti adhesive layer and a 100 nm-thick Pt layer were sequentially deposited using an electron beam (E-beam) evaporator deposition system. Then, to form the RS layer, a 150 nm-thick Ti layer was deposited using an E-beam evaporator. Subsequently, TiO_x_ was formed with MW-assisted oxidation under the following conditions: rated power, 1800 W for 10 min and ambient O_2_, MW frequency, 2.45 GHz. The SPE–chitosan solution was spin-coated on the TiO_x_ layer at 6000 rpm for 30 s, dried in ambient air for 24 h, and oven-baked at 80 °C for 10 min. The thickness of the baked chitosan layer was 80 nm. Finally, a 150 nm-thick and 200 μm diameter Ti top electrode (TE) was deposited onto the RS layer using the E-beam evaporator and a shadow mask. The effect of MW-assisted oxidation was verified by fabricating an SPE–chitosan memristor without the TiO_x_ layer. Figure 1a depicts a schematic diagram of the SPE–chitosan memristor with a TiO_x_ layer developed with MW-assisted oxidation, and Figure 1b,c show optical microscopy images of a SPE–chitosan memristor without and with the TiO_x_ layer, respectively, at a magnification of 150×.

### 2.4. Characterization

During measurement, the SPE–chitosan memristors with and without a TiO_x_ layer developed with MW-assisted oxidation were placed on a two-point probe station within a dark shielded box to mitigate electrical noise and external light interference. The RS characteristics and memristive synaptic functions were measured using an Agilent 4156B Precision Semiconductor Parameter Analyzer (Hewlett-Packard Co., Palo Alto, CA, USA). To verify synaptic modulation behavior, electrical pulse stimulations were applied using an Agilent 8110A Pulse Generator (Hewlett-Packard Co.). The fabricated memristors were imaged under an optical microscope using a Sometech SV-55 microscope system (Seoul, Republic of Korea).

## 3. Results and Discussion

**Figure 2:**
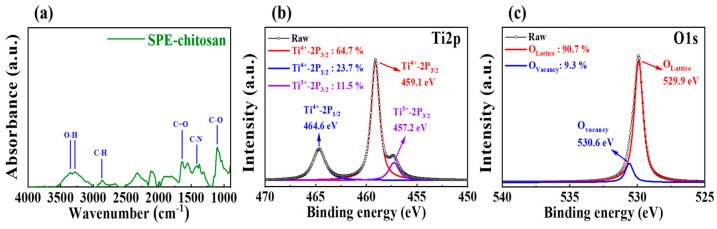
(**a**) FT-IR spectra of the SPE–chitosan film. XPS peaks of Ti2p (**b**) and O1s (**c**) in the TiO_x_ layer developed from MW-assisted oxidation.

Fourier-transform infrared spectroscopy (FT-IR) was used to analyze the chemical composition of the chitosan electrolyte membrane, an insulating layer, prior to estimating the electrical properties and synaptic operation of the memristors. Figure 2a shows the FT-IR spectrum of the chitosan electrolyte film at wavelengths ranging from 4000 to 900 cm^−1^, revealing stretching peaks of O-H and C-H around 3350 and 2860 cm^−1^, respectively. The 1700–1600 cm^−1^ range was primarily influenced by the amide I region, with the peak at approximately 1640 cm^−1^ attributed to C=O stretching. The peaks at 1398 and 1101 cm^−1^ were owing to C-N and C-O stretching, respectively. In general, the spectrum of the chitosan electrolyte film displayed peaks related to amide and -OH groups, which are crucial components of proteins and contribute to the conductivity and mobility of protons [24,25]. Next, we used X-ray photoelectron spectroscopy (XPS) to investigate the Ti2p peak of the embedded TiO_x_ layer. As shown in Figure 2b, the primary peaks in Ti^4+^ of Ti2p_1/2_, Ti^4+^ of Ti2p_3/2_, and Ti^3+^ of Ti2p_3/2_ signals that corresponded with the TiO_x_ component were detected, with binding energies of 464.6, 459.1, and 457.2 eV, respectively. The Ti metallic peak was not observed, indicating nearly complete oxidation of TiO_x_. In Figure 2c, the O1s peak was deconvoluted using two peaks. The peaks at the binding energies of 529.9 and 530.6 eV may be attributed to oxygen bound to the TiO_x_ layer lattice and oxygen ions near the oxygen vacancy (V_o_) in the TiO_x_ layer, respectively. The presence of V_o_ is a key factor in endowing this material system with RS performance [26,27,28].

**Figure 3:**
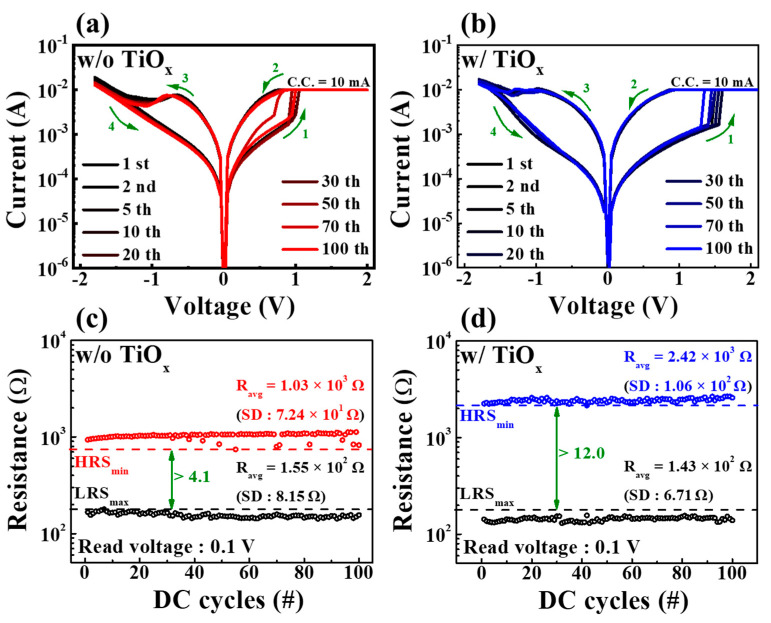
RS endurance characteristics during 100 DC cycles. BRS I–V curves of SPE–chitosan memristors without (**a**) and with (**b**) TiO_x_. Resistance values of SPE–chitosan memristors without (**c**) and with (**d**) TiO_x_.

Figure 3a,b depict the endurance characteristics of SPE–chitosan memristors prepared without and with TiO_x_ during 100 DC cycles. These characteristics were measured by applying DC bias while grounding the BE. When the TE voltage was applied in the positive direction (as indicated by green arrow 1), the memristor entered the SET (ON) state, indicating a change in resistance from a high-resistance state (HRS) to a low-resistance state (LRS). Conversely, when the voltage was swept in the negative direction (as indicated by green arrow 3), the device changed its resistance state from LRS to HRS, resulting in a RESET (OFF) operation. Thus, both SPE–chitosan memristors exhibited typical BRS properties. The contact between the electrode and the chitosan electrolyte can be utilized for cation-based electrochemical conversion owing to the redox reaction of mobile ions in the polymer electrolyte [6,29]. The electrochemical metallization reaction significantly affects the RS operation when an electric field is applied to the electrode of the chitosan–TiO_x_ nanocomposite film. Metal electrodes that are reactive to electrochemistry supply and discharge mobility cations allow the development of highly conductive filaments [30]. The HRS and LRS were extracted from repetitive BRS I–V curves at a read voltage of 0.1 V, as shown in Figure 3c,d. For the SPE–chitosan memristor without TiO_x_, the average resistance values of the HRS and LRS were 1.03 × 10^3^ Ω and 1.55 × 10^2^ Ω, respectively, with standard deviations (SDs) of 7.24 × 10^1^ Ω and 8.15 Ω. In contrast, the average resistance values of the HRS and LRS for the SPE–chitosan memristor with embedded TiO_x_ were 2.42 × 10^3^ Ω and 1.43 × 10^2^ Ω, respectively, with SDs of 1.06 × 10^2^ Ω and 6.71 Ω. This indicates that the SPE–chitosan memristor with TiO_x_ has a larger RS memory window and lower SD than the one without TiO_x_. Furthermore, the RS window, defined as the minimum HRS/maximum LRS, increased from 4.1 to 12.0 due to the embedded TiO_x_. This can be attributed to the abundant oxygen lattice and ions in TiO_x_, leading to a stable RS operation and higher HRS, resulting in a larger memory window [31].

**Figure 4:**
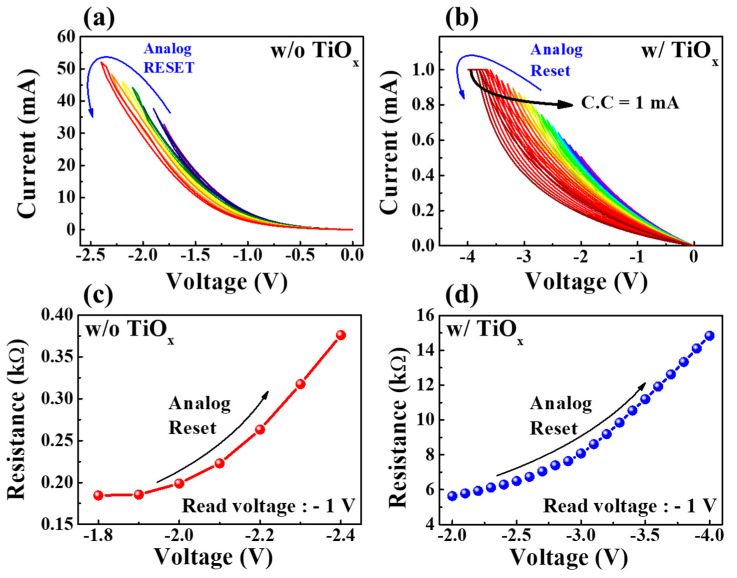
Analog RESET process for SPE–chitosan memristors without (**a**) and with (**b**) TiO_x_. Graphs (**c**,**d**) show the corresponding resistances extracted at a read voltage of −1 V.

Modulating multi-step conduction is a key factor in achieving high-density memory storage for synaptic devices. Figure 4a,b demonstrate the analog RESET process of SPE–chitosan nanocomposite memristors without and with TiO_x_, respectively. The SPE–chitosan memristor without TiO_x_ exhibited seven analog RESET states with a gradually decreasing range of the maximum negative RESET voltage between −1.8 and −2.4 V with an interval of −0.1 V after a single positive digital SET operation. In contrast, the SPE–chitosan memristor with TiO_x_ had 21 analog RESET states with a compliance current of 1 mA, and a RESET voltage range of −2 to −4 V with an interval of −0.1 V after one positive digital SET operation. The storage capacity corresponding to the memory window of the memristor depends on the size of the I_on_/I_off_ changes. The results shown in Figure 4a,b demonstrate that our approach significantly increases the storage capacity density of a memristor device. Figure 4c,d depict the resistance change in each device, extracted at a read voltage of −1 V. Notably, the SPE–chitosan memristor with TiO_x_ exhibited a significantly higher resistance change value (∆9.20 kΩ) than the SPE–chitosan memristor without TiO_x_ (∆0.19 kΩ), indicating more stable and reliable memristive operation. Moreover, the SPE–chitosan–TiO_x_ nanocomposite memristor displayed multi-level changes in resistance, allowing for the representation of multi-level weight changes in synapses. These findings suggest that the SPE–chitosan–TiO_x_ nanocomposite memristor holds great potential for both memristive switching operation and artificial synaptic function [32].

**Figure 5:**
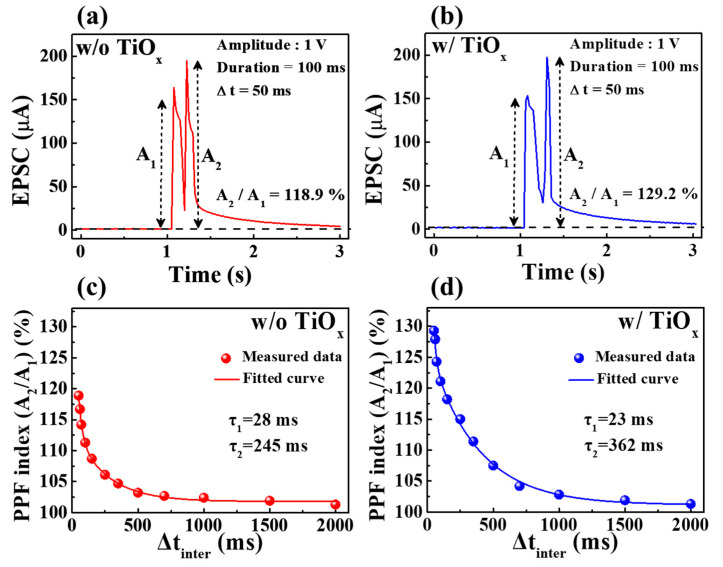
EPSCs triggered by paired presynaptic spikes in SPE–chitosan memristors without (**a**) and with (**b**) TiO_x_. PPF index values of SPE–chitosan memristors without (**c**) and with (**d**) TiO_x_.

Neural facilitation, which is a dynamic increase in a transporter level and the decoding of biological information, such as visual or auditory data, is an important concept in biological neuroscience. PPF is a typical property of short-term synaptic plasticity and a form of neural facilitation, whereby the second pre-synaptic spike amplifies the first post-synaptic spike. Depending on the time interval (∆t_inter_) between two successive pre-synaptic spikes, the second synaptic spike causes a larger excitatory post-synaptic current (EPSC) for PPF. The proton transfer ions moved by the first spike accumulate between the electrolyte and the interface. At short ∆t_inter_, mobile protons accumulate continuously at the interface because they have insufficient time to return to their initial position [33,34]. Figure 5a,b depict the EPSC responses triggered by paired pre-synaptic spikes with an amplitude of 1 V and a duration of 100 ms applied at 50 ms intervals of SPE–chitosan memristors without and with TiO_x_, respectively. Both memristors showed a higher response in the second EPSC (A_2_) than in the first (A_1_), with the SPE–chitosan memristor with TiO_x_ exhibiting a larger EPSC than the one without. The PPF index, calculated as the ratio of the EPSC peak amplitudes (A_2_/A_1_), is shown in Figure 5c,d as a function of ∆t_inter_. The PPF index increases for short ∆t_inter_ and decreases for long ∆t_inter_, mimicking a biological synaptic response [35]. Notably, the SPE–chitosan memristor with TiO_x_ had a higher PPF index value (~129.2%) than the one without (~118.9%). The PPF index values were fitted using a double-exponential decay function [36]:

(1) $$\mathrm{PPF}\;\mathrm{index}=A+C_{1}\exp(-\Delta t/\tau_{1})+C_{2}\exp(-\Delta t/\tau_{2})$$

where A is a constant fixed value, C_1_ and C_2_ indicate the initial facilitation magnitude values, and τ_1_ and τ_2_ represent typical relaxation times, respectively. The exponential decay process of PPF can be well modeled by a double exponential decay relation, as shown by the solid lines in Figure 5c,d [37]. For the SPE–chitosan memristor without TiO_x_, the values of τ_1_ and τ_2_ were 28 ms and 245 ms, respectively, whereas for the memristor with TiO_x_, the values were 23 ms and 362 ms, respectively. These values are in good agreement with those observed in biological synapses and demonstrate that the proposed devices can mimic the fast and slow timescales of synaptic events, which occur on the order of tens and hundreds of milliseconds, respectively [38].

**Figure 6:**
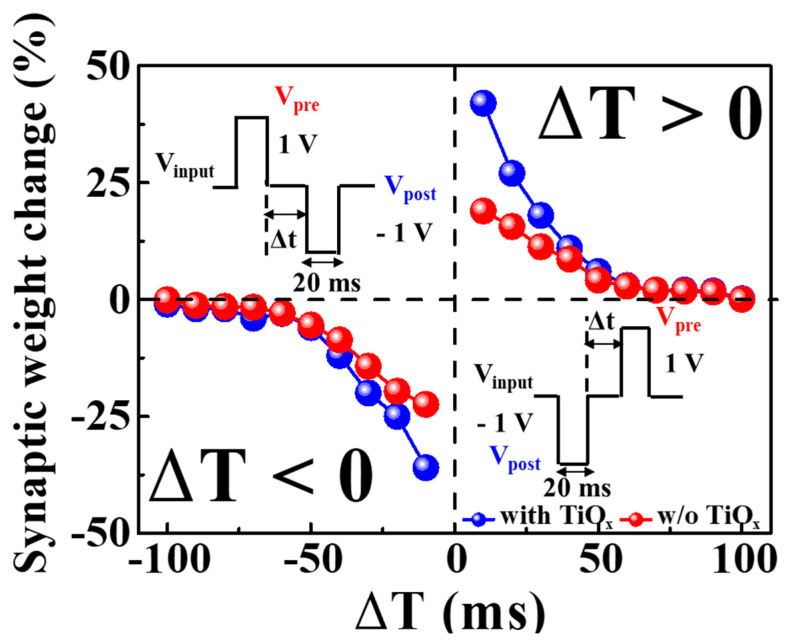
STDP characteristics of excitatory response modes of SPE–chitosan memristors with and without TiO_x_. Insets represent timing differences between pre-synaptic and post-synaptic signals through a series of spikes.

The short-term plasticity of synapses is temporary, whereas long-term plasticity allows for memory through changes in the synaptic weights. STDP is a crucial mechanism for memory and learning in biological neural networks and is determined by the temporal sequence of activity between pre- and post-synaptic neurons. STDP improves on Hebbian learning rules, which regulate neural connection strength, through temporal correlation neural learning. Its simplicity, biological relevance, and computational capabilities in neuroscience make STDP highly interesting [39,40]. Long-term potentiation (LTP) and long-term depression (LTD) in the proposed memristors are determined by STDP and characterized by a constant increase and sustained weakening of synaptic weight, respectively. Figure 6 illustrates the STDP features in the excitatory response mode, wherein the synaptic weight (ΔT = t_post_ − t_pre_) for precise time differences is influenced by the pre-synaptic arrival time (t_pre_) and the post-synaptic production time (t_post_). The synaptic weight change (ΔW) is plotted as dots, showing asymmetric Hebbian learning STDP properties, which resemble biological STDP functions. When the post-synaptic spike follows the pre-synaptic spike (ΔT > 0), long-term potentiation occurs due to the strengthening of the synaptic weights, resulting from a decrease in |∆T|. Conversely, when the post-synaptic spike precedes the pre-synaptic spike (ΔT < 0), long-term depression properties occur due to a weakening of the synaptic weights. Additionally, as |∆T| increases, the synaptic weight change decreases. In the inverted STDP mode, the SPE–chitosan memristor’s response mode becomes inhibitory. The STDP learning function can be defined using the following equation [41,42]:

(2) $$\Delta W=\{\begin{array}{c} A^{+}e^{-\Delta T/\tau^{+}},\;\;\mathrm{if}\;\Delta T>0\\ -A^{-}e^{\Delta T/\tau^{-}},\;\;\mathrm{if}\;\Delta T<0 \end{array}$$

The time constant *τ*^±^ represents the range of ∆T within which synaptic connections can be either strengthened or weakened. When ΔT approaches zero, the maximal synaptic weight change is determined by A^+^. Consequently, the synaptic weight change values (in percentage) of SPE–chitosan memristors with TiO_x_ exceed those of memristors without TiO_x_. These findings suggest that SPE–chitosan memristors with TiO_x_ more accurately emulate the biological STDP operation than those without TiO_x_ [43].

**Figure 7:**
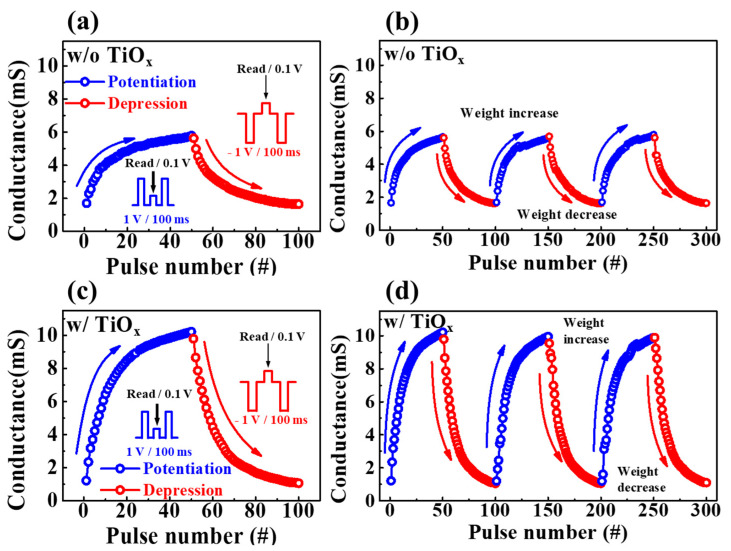
One cycle of 50 potentiation pulses and 50 depression pulses in SPE–chitosan memristors without (**a**) and with (**c**) TiO_x_. Three consecutive cycles of conductance modulation operations were successively performed by applying 300 pulses on SPE–chitosan memristors without (**b**) and with (**d**) TiO_x_.

To investigate the gradual modulation of conductivity corresponding to crucial electrical pulse stimulation for memristive switching, the changes in synaptic weight during potentiation and depression were examined. Figure 7a,c illustrate the characteristics of conductance modulation in response to repetitive pre-synaptic spikes. One cycle of 50 potentiation pulses (1 V/100 ms) and 50 depression pulses (−1 V/100 ms) clearly induced conductivity changes. The inset schematics represent a single pre-spike indicating potentiation and depression read pulses. Figure 7c shows that the dynamic range of conductance modulation for SPE–chitosan memristors with TiO_x_ was approximately 9.7 mS, whereas, for memristors without TiO_x_, it was approximately 5.6 mS, as shown in Figure 7a. The endurance properties for potentiation and depression over three cycles, which indicate the reliability of weight modulation, are depicted in Figure 7b,d. The results demonstrate that SPE–chitosan memristors with TiO_x_ maintain stable operation with a wide conductance dynamic range during cycle repetition, whereas memristors without TiO_x_ exhibit small changes in conductance. Therefore, the proposed SPE–chitosan memristor with TiO_x_ is more effective in improving memory function and achieving uniform weight modulation in response to potentiation and depression pulses. Moreover, the high conductivity of the artificial synaptic device suggests its potential for enhanced learning effects, further validating its feasibility for practical applications [44,45,46].

All conductivity values were normalized to the maximum conductivity (G/G_max_) shown in Figure 7a,c. We determined the dynamic range (DR), asymmetric ratio (AR), and linearity, which are highly correlated with learning and recognition simulation, by examining the nonlinearity of the normalized conductance. The DR (G_max_/G_min_) of the SPE–chitosan memristor with TiO_x_ was 9.64, and the DR of the SPE–chitosan memristor without TiO_x_ was 3.5, which was 2.75 times that of the memristors without TiO_x_. Higher DR values indicate improved precision and recognition performance [47]. The AR represents the asymmetry of changes in conductance potentiation and depression. To evaluate the asymmetry of changes in conductance potentiation and depression, we calculated the AR using Equation (3), where G_p_(n) and G_d_(n) denote the conductance values during the nth pulse of potentiation and depression, respectively [48]. The AR is a key parameter that can provide insights into the learning and recognition performance of neuromorphic computing systems and can aid in optimizing the design of artificial synapses:

(3) $$\mathrm{AR}=\frac{\mathrm{MAX}\left|G_{p}\left(n\right)-G_{d}\left(n\right)\right|}{G_{p}\left(30\right)-G_{d}\left(30\right)}\;\mathrm{for}\;n=1\;\mathrm{to}\;30$$

An AR value closer to 0 indicates a more symmetrical conductivity change, which can lead to optimal learning performance. The AR values for the SPE–chitosan memristors with and without TiO_x_ were 0.7 and 0.72, respectively. The value of the device with TiO_x_ was closer to 0, indicating a more symmetrical conductivity change. Furthermore, the linearity of the conductivity was confirmed by extracting the nonlinearity factor using Equation (4) [49]:

(4) G={{(Gmaxα−Gminα)× w+Gminα}1αGmin×(Gmax/Gmin)w if α≠0, if α=0.

where G_max_ and G_min_ indicate the maximum and minimum conductivity, respectively, and w is an internal variable between 0 and 1. The nonlinearity factor, denoted as α, controls potentiation (α_p_) or depression (α_d_), with an ideal value of 1. The α_p_ and α_d_ values for the SPE–chitosan memristors with and without embedded TiO_x_ were 2.91 and −0.9, respectively, indicating a higher linearity in the conductivity increase and decrease in the memristors with TiO_x_ than in that without TiO_x_ (α_p_ = 4.85_,_ α_d_ = −2.12). The SPE–chitosan memristor with embedded TiO_x_ allows for an efficient control of the conductivity through lower amplitudes and fewer pulse numbers. Therefore, the proposed device enables low-power and high-speed operation and is expected to achieve a more efficient learning effect. Consequently, the SPE–chitosan memristor with embedded TiO_x_ is expected to demonstrate great potential as an artificial synapse for data processing compared to the memristor without TiO_x_.

## 4. Conclusions

We developed a high-performance organic–inorganic hybrid memristor with embedded TiO_x_, which was formed by applying low-thermal-budget MW-assisted oxidation to an SPE–chitosan layer. Two types of memristors, with and without the TiO_x_ layer, were prepared to examine the effect of this layer on the organic–inorganic hybrid memristor synaptic device properties. We characterized their resistive and memristive switching properties, as well as various biological synapse functions. Both devices exhibited BRS behavior, endurance, and consistent resistance distribution over 100 DC cycles. However, the device with the TiO_x_ layer demonstrated a larger memory window owing to the formation of highly conductive filaments at the interface with the chitosan layer through metal ion adsorption. Furthermore, the SPE–chitosan memristor with TiO_x_ demonstrated improved memristive switching operation, effectively emulating short- and long-term synaptic plasticity through phenomena such as PPF, STDP, and potentiation properties. These improvements were attributed to the larger change in synaptic weights than in the device without TiO_x_. In conclusion, the SPE–chitosan memristor with an embedded TiO_x_ layer, which effectively mimics biological artificial synapses, holds great promise for biocompatible and environmentally friendly neuromorphic systems.

## Data Availability

Not applicable.
